# Draft Genome Sequence of Bacillus altitudinis Lc5, a Biocontrol and Plant Growth-Promoting Endophyte Strain Isolated from Indigenous Black Rice of Manipur

**DOI:** 10.1128/genomeA.00601-18

**Published:** 2018-06-28

**Authors:** Momota Potshangbam, Dinabandhu Sahoo, Preveen Verma, Sandhya Verma, Mohan Chandra Kalita, Sarangthem Indira Devi

**Affiliations:** a Microbial Resources Division, Institute of Bioresources and Sustainable Development (IBSD), Department of Biotechnology, Government of India, Imphal, Manipur, India; b Department of Biotechnology, Gauhati University, Guwahati, Assam, India; c National Institute of Plant Genome Research, Jawaharlal Nehru University Campus, New Delhi, India

## Abstract

We report here the 3.6-Mb draft genome of Bacillus altitudinis Lc5, a potential plant growth promoter and an active antagonistic endophyte of black rice. This genome study will provide better insights into the strain’s mechanisms for plant growth promotion and biocontrol, thus facilitating its application in organic agriculture.

## GENOME ANNOUNCEMENT

Bacillus altitudinis Lc5 was isolated by conventional microbial isolation techniques from an effectively sterilized leaf tissue of healthy indigenous black rice of Manipur, India. To our knowledge, this is the first Bacillus altitudinis endophyte strain reported from black rice. The strain could tolerate abiotic stress when grown in different experimental conditions and produce major defensive enzymes and growth-promoting factors like beta-1,3-glucanase, siderophore, protease, chitinase, cellulase, P-solubilizing ability, indole acetic acid, nitrogen-fixing ability, and antagonistic activity against the phytopathogens Fusarium oxysporum, Rhizoctonia solani, Sclerotium oryzae, Pyricularia oryzae, and Pythium ultimun. At present, a total of 14 genome sequences of B. altitudinis strains with various functional activities, including biocontrol and plant growth-promoting traits (1–3), have been deposited in GenBank.

The genome sequencing was performed on the Illumina HiSeq 2500 platform. Libraries were prepared with Illumina technology, and the adapter sequences were removed using Cutadapt version 1.8 (4). All low-quality (Q < 30) data were filtered out using Sickle version 1.33 (5), and the cleaned reads were subjected to KmerGenie (6) to predict the optimal *k* value and assembly size, which were found to be 27 and 3,760,383 bp, respectively. *De novo* assembly was performed using ABySS version 2.0.1 (7). The draft genome assembly obtained after trimming the paired-end reads comprised 91 scaffolds, with a total length of 3,741,201 bp in 91 scaffolds, with a largest contig size of 885,254 bp, a contig *N*_50_ size of 691,681 bp, and a G+C content of 41.89%. Genes were predicted from the ABySS-assembled contigs using Glimmer version 3.02 (8). The number of predicted genes was 3,950, and the number of genes with a significant BLASTx match (E value ≤ 1 × 10^−5^ and similarity score ≥40%) was 3,286. The NCBI Prokaryotic Genome Annotation Pipeline (PGAP) (https://www.ncbi.nlm.nih.gov/genome/annotation_prok) was used for automatic annotation and prediction of the assembled contigs, which predicted 69 contigs (*L*_50_ = 6), 3,824 genes, 3,679 protein-coding sequences, 74 tRNAs, 24 rRNAs, and 145 pseudogenes.

Annotation of the Lc5 genome revealed several genes and biosynthetic pathways, including antioxidant enzymes, such as catalase, peroxidase, and superoxide dismutase, that support the strain’s potential role as a biocontrol agent against factors such as temperature stress, salinity stress, drought stress, and oxidative stress. Also present in the annotation were gene clusters and pathways related to phosphorous solubilization, iron uptake, cellulose degradation, chitinolytic activity, glucanase, acetoin dehydrogenase, protease, trehalose metabolism, exopolysaccharides, cytokinin, and tryptophan biosynthesis, as well as nitrogen-fixing proteins and genes responsible for sporulation, motility, chemotaxis, and quorum sensing.

Antibiotics and secondary metabolites were analyzed using antiSMASH version 4.1.0 (9). Also predicted were 26 gene cluster types comprising a terpene-siderophore, type III polyketide synthase, bacteriocin, nonribosomal peptide synthetase, sactipeptide, and biosynthetic gene clusters of bacilysin, pseudomonine, fengycin, surfactin, and lichenysin. Thus, we conclude that B. altitudinis Lc5 harbors important putative genes, and further exploration will lead to significant contributions toward its sustainable application in organic agricultural practices.

### Accession number(s).

This whole-genome shotgun project has been deposited at DDBJ/ENA/GenBank under the accession no. QCWN00000000. The version described in this paper is the first version, QCWN01000000.

## References

[1] Kumaravel S, Thankappan S, Raghupathi S, Uthandi S 2018 Draft genome sequence of plant growth-promoting and drought-tolerant *Bacillus altitudinis* FD48, isolated from rice phylloplane. Genome Announc 6(9):e00019-18. doi:10.1128/genomeA.00019-18. 29496824 PMC5834328

[2] Chen Y, Rekha P, Arun A, Shen F, Lai W-A, Young C 2006 Phosphate solubilizing bacteria from subtropical soil and their tricalcium phosphate solubilizing abilities. Appl Soil Ecol 34:33–41. doi:10.1016/j.apsoil.2005.12.002.

[3] Budiharjo A, Jeong H, Wulandari D, Lee S, Ryu C-M 2017 Complete genome sequence of *Bacillus altitudinis* P-10, a potential bioprotectant against *Xanthomonas oryzae* pv. oryzae, isolated from rice rhizosphere in Java, Indonesia. Genome Announc 5(48):e01388-17. doi:10.1128/genomeA.01388-17. PMC5722074 29192088

[4] Martin M 2011 Cutadapt removes adapter sequences from high-throughput sequencing reads. EMBnet J 17:10–17.

[5] Joshi N, Fass J 2011 Sickle: a sliding-window, adaptive, quality-based trimming tool for FastQ files, version 1.33. https://github.com/najoshi/sickle.

[6] Chikhi R, Medvedev P 2014 Informed and automated *k*-mer size selection for genome assembly. Bioinformatics 30:31–37. doi:10.1093/bioinformatics/btt310. 23732276

[7] Jackman SD, Vandervalk BP, Mohamadi H, Chu J, Yeo S, Hammond SA, Jahesh G, Khan H, Coombe L, Warren RL, Birol I 2017 ABySS 2.0: resource-efficient assembly of large genomes using a Bloom filter. Genome Res 27:768–777. doi:10.1101/gr.214346.116. 28232478 PMC5411771

[8] Delcher AL, Bratke KA, Powers EC, Salzberg SL 2007 Identifying bacterial genes and endosymbiont DNA with Glimmer. Bioinformatics 23:673–679. doi:10.1093/bioinformatics/btm009. 17237039 PMC2387122

[9] Weber T, Blin K, Duddela S, Krug D, Kim HU, Bruccoleri R, Lee SY, Fischbach MA, Müller R, Wohlleben W, Breitling R, Takano E, Medema MH 2015 antiSMASH 3.0—a comprehensive resource for the genome mining of biosynthetic gene clusters. Nucleic Acids Res 43:W237–W243. doi:10.1093/nar/gkv437. 25948579 PMC4489286

